# δ‐Tocotrienol induces apoptosis, involving endoplasmic reticulum stress and autophagy, and paraptosis in prostate cancer cells

**DOI:** 10.1111/cpr.12576

**Published:** 2019-02-04

**Authors:** Fabrizio Fontana, Roberta Manuela Moretti, Michela Raimondi, Monica Marzagalli, Giangiacomo Beretta, Patrizia Procacci, Patrizia Sartori, Marina Montagnani Marelli, Patrizia Limonta

**Affiliations:** ^1^ Department of Pharmacological and Biomolecular Sciences Università degli Studi di Milano Milano Italy; ^2^ Department of Environmental Science and Policy Università degli Studi di Milano Milano Italy; ^3^ Department of Biomedical Sciences for Health Università degli Studi di Milano Milano Italy

**Keywords:** apoptosis, autophagy, ER stress, paraptosis, prostate cancer, δ‐tocotrienol

## Abstract

**Objectives:**

Prostate cancer, after the phase of androgen dependence, may progress to the castration‐resistant prostate cancer (CRPC) stage, with resistance to standard therapies. Vitamin E‐derived tocotrienols (TTs) possess a significant antitumour activity. Here, we evaluated the anti‐cancer properties of δ‐TT in CRPC cells (PC3 and DU145) and the related mechanisms of action.

**Materials and methods:**

MTT, Trypan blue and colony formation assays were used to assess cell viability/cell death/cytotoxicity. Western blot, immunofluorescence and MTT analyses were utilized to investigate apoptosis, ER stress and autophagy. Morphological changes were investigated by light and transmission electron microscopy.

**Results:**

We demonstrated that δ‐TT exerts a cytotoxic/proapoptotic activity in CRPC cells. We found that in PC3 cells: (a) δ‐TT triggers both the endoplasmic reticulum (ER) stress and autophagy pathways; (b) autophagy induction is related to the ER stress, and this ER stress/autophagy axis is involved in the antitumour activity of δ‐TT; in autophagy‐defective DU145 cells, only the ER stress pathway is involved in the proapoptotic effects of δ‐TT; (c) in both CRPC cell lines, δ‐TT also induces an intense vacuolation prevented by the ER stress inhibitor salubrinal and the protein synthesis inhibitor cycloheximide, together with increased levels of phosphorylated JNK and p38, supporting the induction of paraptosis by δ‐TT.

**Conclusions:**

These data demonstrate that apoptosis, involving ER stress and autophagy (in autophagy positive PC3 cells), and paraptosis are involved in the anti‐cancer activity of δ‐TT in CRPC cells.

## 1 INTRODUCTION

Prostate cancer (PCa) represents the third cause of cancer‐related deaths among men in the Western countries.^1^ Androgen‐deprivation therapy (GnRH agonists, either alone or in combination with an antiandrogen) represents the treatment of choice for this pathology.^2^, ^3^ However, the tumour frequently progresses towards a condition of castration resistance (castration‐resistant prostate cancer, CRPC) for which the therapeutic options are limited.^5^, ^6^

Several natural compounds were reported to possess anti‐cancer properties^7^ by inducing apoptosis, through modulation of intracellular pathways including endoplasmic reticulum (ER) stress and autophagy.^8^, ^9^

Cells react to severe stress conditions by accumulating misfolded proteins in the ER, leading to dissociation from the chaperone BiP (immunoglobulin heavy‐chain‐binding protein, GRP78) and activation of three stress sensors: PERK (double‐stranded RNA‐dependent protein kinase PKR‐like ER kinase), ATF6 (activating transcription factor 6) and IRE1α (inositol‐requiring enzyme 1α). Each of these proteins couples to a specific intracellular pathway converging to apoptosis.^14^, ^15^ The transcription factor CHOP (C/EBP homologous protein, also known as GADD153) plays a key role in the ER stress‐related apoptosis pathway.

Autophagy consists of the degradation/recycling of damaged cytoplasmic proteins and organelles that are sequestered in autophagosomes and then degraded in autophagolysosomes. Several markers of the autophagic phases, such as LC3‐II (the phosphatidylethanolamine‐conjugated form of LC3 microtubule‐associated protein 1AB‐light chain), and sequestosome 1/p62 (SQSTM1/p62)^16^ were identified. Autophagy is involved both in suppression of cancer growth and in development of drug resistance in cancer cells.^17^ There is now evidence connecting the ER stress‐autophagy axis with apoptosis, representing a molecular target of cancer treatments.^8^

Non‐canonical cell death mechanisms are also involved in natural compounds anti‐cancer activity.^18^, ^19^ Paraptosis is a programmed cell death characterized by cytoplasmic vacuolation resulting from mitochondrial and/or ER swelling and requiring protein synthesis.^21^

Vitamin E is composed of two groups of compounds, tocopherols (TPs) and tocotrienols (TTs); each group consists of four isomers: α, β, γ and δ. TTs (but not TPs) have attracted interest for their health‐promoting properties, such as anti‐cancer activity. δ‐ and γ‐TT were reported to exert anti‐cancer activities through the modulation of different intracellular pathways.^22^, ^23^ The bioavailability/safety of these compounds was demonstrated in healthy subjects^25^, ^26^ and in cancer patients.^27^

Here, we dissected the antitumour effects of δ‐TT in CRPC cells by exploring the involvement of the ER stress‐autophagy axis in this activity; we also investigated the possible involvement of non‐canonical programmed cell deaths (ie paraptosis) in its effects.

## 2 MATERIALS AND METHODS

### 2.1 Chemicals

δ‐TT was purified from a commercial extract of Annatto (*Bixa orellana* L.) seeds (American River Nutrition Inc, Hadley, MA, USA).^28^

Primary antibodies against: caspase 3 (9656), cleaved caspase 3 (9664), PARP (9542), BiP (3177), eIF2α (5324), p‐eIF2α (3398), ATF4 (11815), CHOP (2895), IRE1α (3294), PDI (3501) were from Cell Signaling Technology Inc, Boston, MA, USA; SQSTM1/p62 (PA5‐20839) was from Thermo Fisher Scientific, Rodano, Milano, Italy; LC3 (L8918); JNK, p38 and α‐tubulin (T6199) were from Sigma‐Aldrich, Milano, Italy, and cytochrome *c* (sc‐13560) was from Santa Cruz Biotechnology Inc, Santa Cruz, CA, USA. Horseradish peroxidase‐conjugated secondary antibody and enhanced chemiluminescence reagents were from Cyanagen (Bologna, Italy). Alexa Fluor 488 and 594 secondary antibodies were from Thermo Fisher Scientific.

Z‐VAD‐FMK (the pan‐caspase inhibitor; FMK001) was from R&D System Inc (Minneapolis, MN). The ER stress inhibitors salubrinal (S) and 4‐PBA (4‐phenylbutyrate), the autophagy inhibitors CQ (chloroquine) and Baf (bafilomycin), the translation inhibitor cycloheximide, and analytical grade solvents were from Sigma‐Aldrich; 3‐MA (3‐methyladenine) was from Selleckchem (Munich, Germany).

### 2.2 Cell lines and cell culture

Normal prostate epithelial RWPE‐1 (provided by Dr N. Zaffaroni; IRCCS, National Institute of Cancer, Milano, Italy) and cancer (DU145 and PC3) cell lines were from American Type Culture Collection (ATCC, Manassas, VA, USA). RWPE‐1 cells were cultured in keratinocyte‐SFM medium supplemented with Bovine Pituitary Extracts and EGF (2.5 μM) (Thermo Fisher Scientific), DU145 and PC3 cells in RPMI medium supplemented with FBS (7.5% and 5% respectively), glutamine and antibiotics. Cells were cultured in humidified atmosphere of 5% CO_2_/95% air at 37°C.

### 2.3 MTT viability assay

Cells were seeded at a density of 3 × 10^4^ cells/well in 24‐well plates for 24 hours and then exposed to the specific compounds. After each treatment, cell viability was determined by 3‐(4,5‐dimethylthiazole‐2‐yl)‐2,5‐diphenyltetrazolium bromide (MTT) assay, as described.^29^

### 2.4 Trypan blue exclusion assay

Cells were plated (5 × 10^4^ cells/dish) in 6‐cm dishes. After 48 hours, cells were treated with δ‐TT (5‐20 μg/mL, 24 hours). Adherent (viable) and floating (dead) cells were harvested, stained with Trypan blue 0.4% (1:1 v/v) and counted by Luna automated cell counter (Logos Biosystems, Annandale, VA, USA).

### 2.5 Colony formation assay

Cells were seeded (100‐250 cells/well, depending on the cell type) in 6‐well plates. After each treatment, a colony formation assay was performed to assess dimensions and numbers of colonies. Colonies were fixed with 70% methanol and stained with Crystal Violet 0.15%. Images of stained colonies were captured by a Nikon photo camera.

### 2.6 Western blot assay

Cells were seeded at 5 × 10^5^ cells/dish in 10‐cm dishes. After each treatment, cells were lysed in RIPA buffer; protein preparations (15‐40 μg) were resolved on SDS‐PAGE and transferred to nitrocellulose (or PVDF for the Western blot of LC3) membranes. Membranes were incubated with the specific primary antibodies. Detection was done using horseradish peroxidase‐conjugated secondary antibodies and enhanced chemiluminescence (Westar Etac Ultra 2.0, XLS075,0100; Cyanagen Srl). Tubulin was utilized as a loading control.

### 2.7 Immunofluorescence assay

Cells were seeded at 3 × 10^4^ cells/well in 24‐well plates on polylysine‐coated 13‐mm coverslips for 48 hours before treatments. After each treatment, cells were fixed and stained with the specific primary antibodies, followed by secondary antibodies. Labelled cells were examined under a Zeiss Axiovert 200 microscope with a 63 × 1.4 objective lens linked to a Coolsnap Es CCD camera (Roper Scientific‐Crisel Instruments, Roma, Italy).

### 2.8 Morphological analysis

Cells were seeded at 3 or 4 × 10^4^ cells/dish in 6‐cm dishes, respectively, and treated with δ‐TT (15 μg/mL for 18 hours). Cytoplasmic vacuolation was analysed by light microscopy from different fields under a Zeiss Axiovert 200 microscope with a 32 × 0.4 objective lens linked to a Coolsnap Es CCD camera (Roper Scientific‐Crisel Instruments). For TEM analysis, cell pellets were fixed over night in a solution containing 2% of paraformaldehyde and 2% glutaraldehyde in 0.1 M sodium cacodylate buffer (pH 7.3). Samples were post‐fixed in 1% osmium tetroxide in cacodylate buffer at 0°C for 90 minutes, washed, dehydrated and embedded in Epon‐Araldite resin. Ultrathin sections were cut by a Leica Supernova ultramicrotome (Reichert Ultracut E) and stained with lead citrate. TEM was performed with a Zeiss EM10 electron microscope.

### 2.9 Statistical analysis

Statistical analysis was performed with a statistic package (GraphPad Prism5, GraphPad Software San Diego, CA, USA). Data are represented as the mean ± SEM of three‐four independent experiments. Differences between groups were assessed by one‐way analysis of variance (ANOVA) followed by Dunnet's or Bonferroni's test. A *P* value <0.05 was considered statistically significant.

## 3 RESULTS

### 3.1 δ‐TT reduces cells viability and exerts a cytotoxic effect in prostate cancer cells

**Figure 1:**
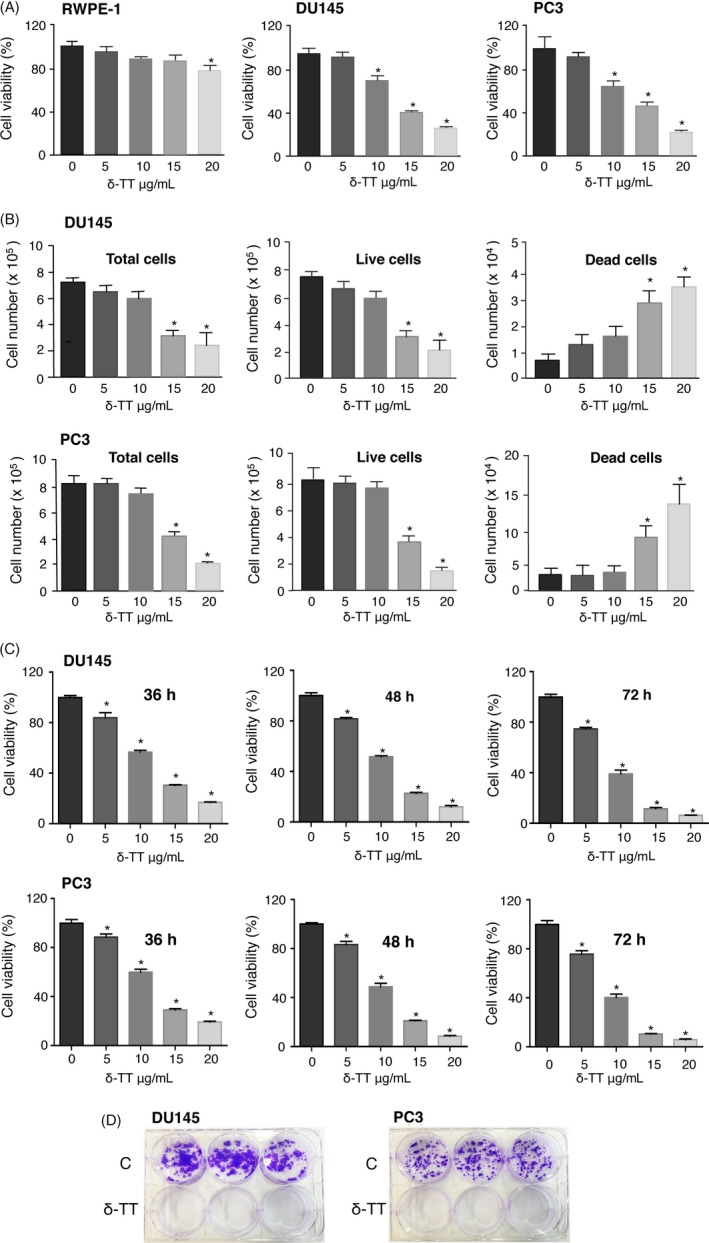
δ‐TT decreases cell viability and exerts a cytotoxic effect on DU145 and PC3 prostate cancer cells. A, RWPE‐1 normal epithelial prostate cells and DU145 and PC3 prostate cancer cells were treated with δ‐TT (5‐20 μg/mL) for 24 h. Cell viability was then evaluated by MTT assay. The IC_50_ values were 2.91 × 10^−5^ M and 3.22 × 10^−5^ M for DU145 and PC3 cells, respectively. B, Prostate cancer cells were treated with δ‐TT (5‐20 μg/mL) for 24 h. Total, live and dead cells were evaluated by Trypan blue exclusion assay. C, DU145 and PC3 cancer cells were treated with δ‐TT (15 μg/mL) for 36‐72 h. Cell viability was then evaluated by MTT assay. D, Prostate cancer cells were treated with δ‐TT (15 μg/mL) for 48 h and then, after withdrawal of the treatment, were left to grow for 11‐12 d, dependently on the cell line‐specific proliferation rate. A colony formation assay was performed to evaluate the ability of the cells to form proliferating colonies (dimensions of colonies) and the survival of colony‐forming cells (number of colonies). Each experiment was repeated three times. Data in (A‐C) represent mean values ± SEM and were analysed by Dunnet's test after one‐way analysis of variance. ^*^ *P* < 0.05 vs 0, controls (vehicle)

DU145 and PC3 or normal RWPE‐1 prostate epithelial cells were treated with δ‐TT (5‐20 μg/mL, 24 hours); cell viability was measured (MTT assay). δ‐TT decreased the number of viable CRPC cells in a dose‐dependent way being significantly effective at 10‐20 μg/mL. The IC_50_ values were 2.91 × 10^−5^ M and 3.22 × 10^−5^ M for DU145 and PC3 cells, respectively. The same treatment affected the growth of RWPE‐1 cells only slightly and at the highest dose (20 μg/mL; Figure 1A).

CRPC cells were treated with δ‐TT (5‐20 μg/mL, 24 hours), then dying (floating) and living (adherent) cells were harvested, stained with Trypan blue and counted. In both cell lines, δ‐TT significantly, and dose‐dependently, decreased the number of viable cells and increased the number of dead cells (Figure 1B).

To obtain growth curve kinetics beyond 24 hours, CRPC cells were treated with δ‐TT (15 μg/mL, 36‐72 hours); cell viability was then measured. δ‐TT significantly and dose‐dependently decreased the number of viable cells at each time point (Figure 1C), confirming results reported in Figure 1A.

The cytotoxic activity of δ‐TT was investigated by colony formation assay. CRPC cells were treated with δ‐TT (15 μg/mL, 48 hours) and left to grow for 11‐12 days in the absence of the treatment, according to the cell line‐specific proliferation rate. We analysed (a) the ability of the cells to form colonies (dimensions of colonies) and (b) the survival of colony‐forming cells (number of colonies). We observed that untreated cells grew forming colonies while none of δ‐TT‐treated cells survived to the treatment; moreover, the ability of the cells to form colonies was prevented by the treatment, supporting a cytotoxic effect of δ‐TT (Figure 1D).

### 3.2 δ‐TT triggers apoptosis in prostate cancer cells

**Figure 2:**
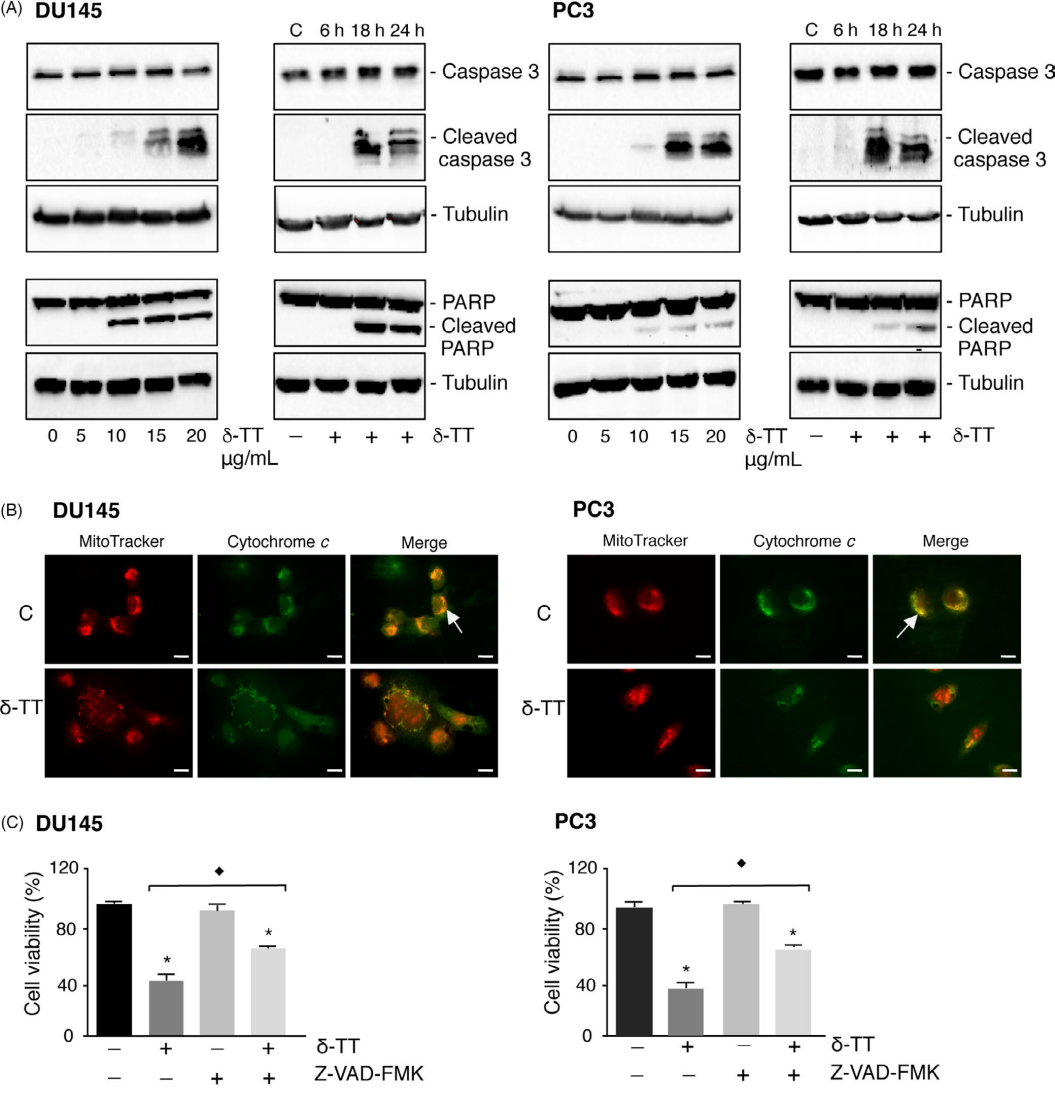
δ‐TT triggers apoptosis in DU145 and PC3 prostate cancer cells. A, DU145 and PC3 cells were treated with δ‐TT (5‐20 μg/mL) for 24 h (left panels) or with δ‐TT (15 μg/mL) for 6‐24 h (right panels). Western blot analysis was carried out to analyse the expression levels of cleaved caspase 3 (ie active) and PARP. Tubulin expression was evaluated as a loading control. 0 and C, controls (vehicle). One representative of three different experiments, for each of the analyses performed, is shown. B, DU145 and PC3 cells were treated with δ‐TT (15 μg/mL) for 18 h; the intracellular localization of cytochrome *c* was then evaluated by immunofluorescence analysis. One representative of three experiments performed is shown. Scale bars are 20 μm. The arrow indicates the cytochrome *c*‐mitochondrial colocalization in controls cells. C, To confirm the involvement of apoptosis in the antitumour activity of δ‐TT, DU145 and PC3 cells were treated with the pan‐caspase inhibitor Z‐VAD‐FMK (50 μM) for 4 h before the tocotrienol (15 μg/mL for 24 h). Cell viability was then evaluated by MTT assay. Each experiment was repeated three times. Data represent mean values ± SEM and were analysed by Bonferroni's test after one‐way analysis of variance. ^*^ *P* < 0.05 vs controls (vehicle). ^♦^ *P* < 0.05 vs δ‐TT‐treated cells

DU145 and PC3 cells were treated with δ‐TT (5‐20 μg/mL, 24 hours); the levels of active (cleaved) caspase 3 were increased by δ‐TT treatment (15 and 20 μg/mL), while cleaved PARP levels increased at the doses of 10‐20 μg/mL, in both cell lines (Figure 2A, left panels). Moreover, active caspase 3 and PARP levels increased in both cell lines at 18‐24 h of treatment (15 μg/mL) (Figure 2A, right panels).

Immunofluorescence studies were performed to confirm the involvement of intrinsic apoptosis in the activity of δ‐TT (15 μg/mL, 18 hours). It was observed that, after treatment, cytochrome *c* was diffused in the cytosol and no overlapping with mitochondria could be observed demonstrating its release from mitochondria (Figure 2B).

CRPC cells were treated with Z‐VAD‐FMK, the pan‐caspase inhibitor (50 μM, 4 hours) before the treatment with δ‐TT (15 μg/mL, 24 hours). Cell viability was significantly reduced by δ‐TT. Z‐VAD‐FMK, given alone, did not modify cell viability; however, pre‐treatment of both cell lines with Z‐VAD‐FMK significantly (even if not completely) reverted the antitumour effect of δ‐TT (Figure 2C).

### 3.3 δ‐TT triggers ER stress in prostate cancer cells

**Figure 3:**
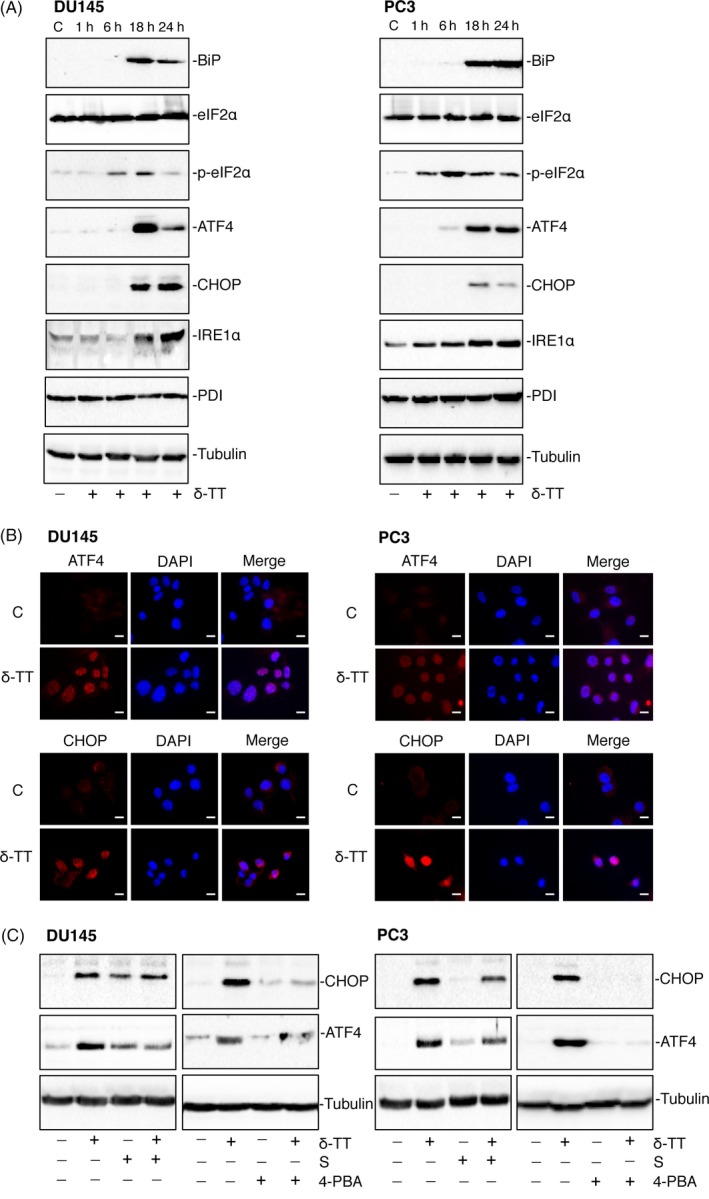
δ‐TT triggers ER stress in DU145 and PC3 prostate cancer cells. A, DU145 and PC3 cells were treated with δ‐TT (15 μg/mL) for 1‐24 h. Western blot analysis was performed to investigate the expression levels of ER stress‐related proteins (BiP, eIF2α, p‐ eIF2α, ATF4, CHOP, IRE1α, PDI). Tubulin expression was evaluated as a loading control. B, DU145 and PC3 cells were treated with δ‐TT (15 μg/mL) for 18 h. The expression levels and intracellular localization of the key transcription factors involved in the ER stress‐mediated apoptosis (ATF4 and CHOP) were evaluated by immunofluorescence analysis. C, controls (vehicle). Scale bars are 20 μm. C, CRPC cells were pretreated with the ER stress inhibitors salubrinal (S; 20 μM) or 4‐PBA (2 mM), for 4 and 1 h, respectively, before treatment with δ‐TT (15 μg/mL) for 24 h. The effects of the treatments were analysed on CHOP and ATF expression levels by Western blot. Tubulin expression was evaluated as a loading control. One representative of three different experiments performed is shown

CRPC cells were treated with δ‐TT (15 μg/mL, 1‐24 hours). The expression of ER stress markers (BiP, eIF2α, p‐eIF2α, IRE1α, PDI) and markers of ER stress‐related apoptosis (ATF4 and CHOP) were analysed by Western blotting. δ‐TT increased the levels of BiP (18‐24 hours) in DU145 and PC3 cells. The levels of p‐eIF2α (but not eIF2α) increased in DU145 (6‐18 hours) and PC3 cells (1‐24 hours). The expression of IRE1α was increased at 18‐24 hours in DU145 and at 1‐24 hours in PC3 cells. On the other hand, the levels of the chaperone protein PDI was unaffected in both cell lines. Finally, ATF4 levels were increased at 18‐24 hours in DU145 cells and at 6‐24 hours in PC3 cells, while those of CHOP increased at 18‐24 hours in both CRPC cell lines (Figure 3A).

The intracellular localization of the transcription factors involved in the ER stress‐mediated apoptosis was analysed in CRPC cells treated with δ‐TT (15 μg/mL, 18 hours) by immunofluorescence. In untreated cells, the levels of ATF4 and CHOP were almost undetectable in both cells lines (confirming the results obtained by Western blot). δ‐TT treatment triggered the expression of these transcription factors together with their nuclear localization (overlapping staining between TRITC‐conjugated antibodies and DAPI; Figure 3B).

To confirm the specificity of the effects of δ‐TT on ER stress‐related proteins, CRPC cells were treated with the tocotrienol (15 μg/mL, 24 hours), either in the absence or in the presence of two ER stress inhibitors: salubrinal (S, 20 μM) or 4‐PBA (2 mM), for 4 and 1 hours, respectively. Figure 3C confirms that δ‐TT induces the expression of CHOP and ATF4 (as in Figure 3A); pre‐treatment with both ER stress inhibitors significantly reduced the expression of both proteins. These results support that δ‐TT triggers ER stress in CRPC cells.

### 3.4 ER stress mediates the antitumour activity of δ‐TT in prostate cancer cells

**Figure 4:**
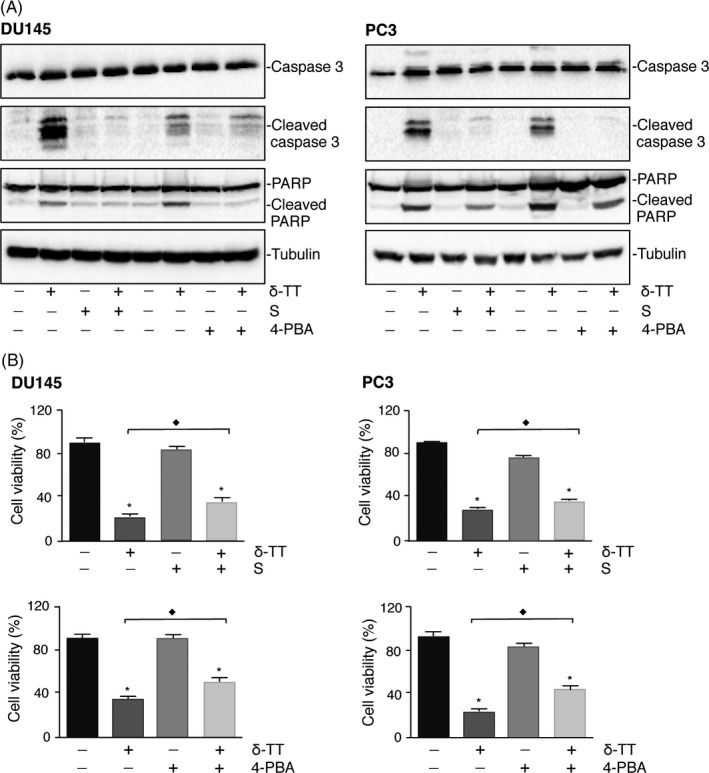
ER stress mediates the proapoptotic activity of δ‐TT in DU145 and PC3 prostate cancer cells. DU145 and PC3 cells were pretreated with the ER stress inhibitors salubrinal (S; 20 μM) or 4‐PBA (2 mM), for 4 and 1 h, respectively, before treatment with δ‐TT (15 μg/mL) for 24 h. A, The expression levels of cleaved (ie active) caspase 3 and PARP were evaluated by Western blot analysis. Tubulin expression was evaluated as a loading control. One representative of three different experiments performed is shown. B, Cell viability was assessed by MTT assay. Each experiment was repeated three times. Data represent mean values ± SEM and were analysed by Bonferroni's test after one‐way analysis of variance. ^*^ *P* < 0.05 vs controls (vehicle). ^♦^ *P* < 0.05 vs δ‐TT‐treated cells

Data from the literature support that ER stress is involved in the antitumour activity of δ‐TT in cancer cells.^29^, ^30^ To confirm this hypothesis in prostate cancer cells, DU145 and PC3 cells were treated with two ER stress inhibitors: salubrinal (20 μM) or 4‐PBA (2 mM), for 4 and 1 hours, respectively, before δ‐TT treatment (15 μg/mL, 24 hours). δ‐TT markedly increased the expression levels of cleaved caspase 3 and PARP, confirming the results reported in Figure 2A. Salubrinal and 4‐PBA, given alone, did not modify the expression of these proteins; however, they significantly counteracted the effects of the tocotrienol on the expression of the cleaved forms of both caspase 3 and PARP (Figure 4A).

Figure 4B shows that cell viability was significantly suppressed by δ‐TT treatment. Salubrinal and 4‐PBA alone did not influence the viability of cancer cells; however, they significantly reverted (although not completely) the cytotoxic activity of δ‐TT, in both cell lines. These results demonstrate that ER stress is involved in the anti‐cancer activity of δ‐TT.

### 3.5 δ‐TT triggers autophagy in PC3 prostate cancer cells

**Figure 5:**
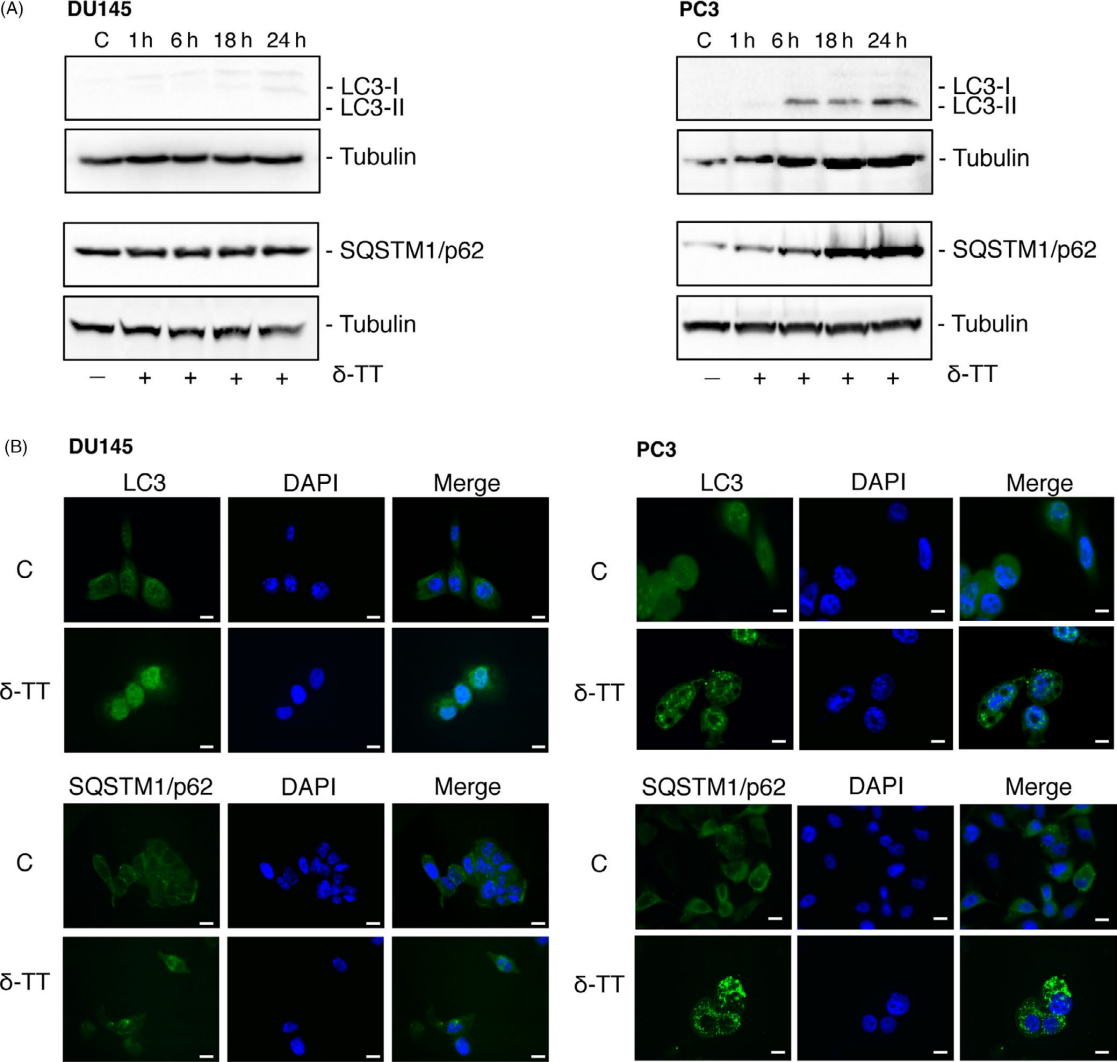
δ‐TT triggers autophagy in PC3, but not in DU145, prostate cancer cells. DU145 and PC3 cells were treated with δ‐TT (15 μg/mL) for 1‐24 h. A, Western blot analysis was performed to investigate the expression levels of autophagy‐related proteins (LC3‐II/LC3‐I, SQSTM1/p62). Tubulin expression was evaluated as a loading control. B, The expression levels and intracellular localization of LC3 and SQSTM1/p62 were evaluated by immunofluorescence. One representative of three different experiments performed is shown. C, controls (vehicle). Scale bars are 20 μm

To assess whether δ‐TT might trigger the autophagy pathway in CRPC cells, DU145 and PC3 cells were treated with δ‐TT (15 μg/mL, 1‐24 hours). We demonstrated that the tocotrienol markedly increases the expression levels of the autophagy‐related proteins LC3‐II (increased LC3‐II/LC3‐I ratio) and SQSTM1/p62 in PC3 cells (at 6‐24 and 1‐24 hours time intervals, respectively) but not in DU145 cells (Figure 5A). In line with this observation, by immunofluorescence analysis, we observed that, in basal conditions, LC3 is poorly expressed in both cell lines; δ‐TT induced the cytoplasmic accumulation of LC3 (LC3 puncta) and p62 bodies formation in PC3 but not in DU145 cells (Figure 5B). These results are in agreement with data reporting that DU145 cells are autophagy‐defective due to an alternative splicing of ATG5 transcript and lack of a full‐length ATG5 protein.^31^ Thus, further studies investigating the involvement of autophagy in δ‐TT anti‐cancer activity were performed in PC3 cells.

### 3.6 δ‐TT triggers ER stress‐related autophagy in PC3 prostate cancer cells

**Figure 6:**
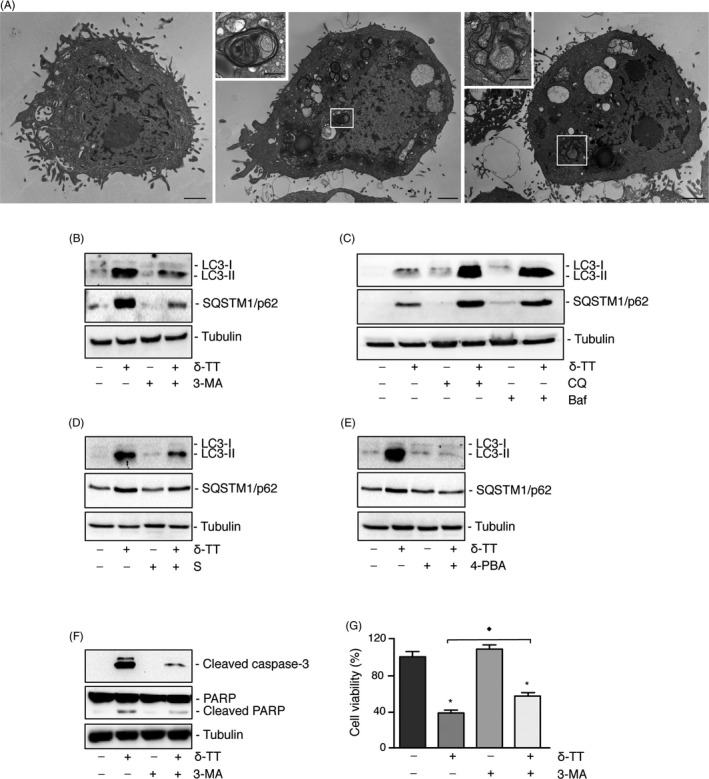
Autophagy, related to ER stress, mediates the antitumour activity of δ‐TT in PC3 prostate cancer cells. A, Cells were treated with δ‐TT (15 μM) for 18 h and TEM images were selected. Left panel is a TEM image of control cells. Boxed area indicates the presence, in δ‐TT‐treated cells, of autophagosomes (middle panel) that can localize at the lysosomal level forming autophagolysosomes (right panel). Scale bars are 2 and 0.7 μm for photographs in boxed areas. B and C, Cells were treated with δ‐TT (15 μg/mL) for 24 h in the presence of either the inhibitor of early stage autophagy 3‐MA (10 mM) (B) or the inhibitors of late stage autophagy CQ (10 μM) and Baf (10 nM) (C). The LC3‐II/LC3‐I ratio and SQSTM1/p62 levels were evaluated by Western blot analysis. D and E, Cells were pretreated with the two ER stress inhibitors salubrinal (S; 20 μM) for 4 h, or 4‐PBA (2 mM) for 1 h, and then with δ‐TT (15 μg/mL) for 18 h. The LC3‐II/LC3‐I ratio and SQSTM1/p62 levels were evaluated by Western blot analysis. F and G, PC3 cells were pretreated with 3‐MA (10 mM) for 4 h before tocotrienol treatment (15 μg/mL) for 24 h. The effects of the treatment were analysed on the expression of apoptosis‐related markers, by Western blot (F) as well as on cell viability, by MTT assay (G). For Western blot analyses, one representative of three different experiments performed is shown. For MTT assay, each experiment was repeated three times and data represent mean values ± SEM and were analysed by Bonferroni's test after one‐way analysis of variance. ^*^ *P* < 0.05 vs C, controls (vehicle). ^♦^ *P* < 0.05 vs δ‐TT‐treated cells

To confirm the activation of an autophagic flux in PC3 cells, we investigated the presence of autophagosomes in δ‐TT‐treated (15 μg/mL, 18 hours) cells by TEM. Figure 6A shows that, at variance with control cells (left panel), autophagosomes containing entire organelles surrounded by multilamellar membranes are present in tocotrienol‐treated cells (middle panel, boxed area) and localize at the lysosomal level forming autophagolysosomes containing remnants of digested structures (right panel, boxed area).

Moreover, cells were pretreated with 3‐MA (10 mM), or with CQ (10 μM) or Baf (10 nM) and then with the tocotrienol (15 μg/mL, 24 hours). Pre‐treatment of the cells with 3‐MA (inhibitor of early stage autophagy) inhibited LC3‐II expression (decreasing the LC3‐II/LC3‐I ratio; Figure 6B); on the contrary, CQ and Baf (inhibitors of the late phase of autophagy) significantly potentiated the effect of δ‐TT on the accumulation of LC3‐II (LC3‐II/LC3‐1 ratio; Figure 6C).

Similar results were obtained on the expression levels of SQSTM1/62 (Figure 6B,C). Normally, the activation of autophagy determines a decrease in the expression of SQSTM1/p62, because of its accumulation in autophagosomes and the final degradation into lysosomes. However, SQSTM1/p62 upregulation, and at least transient increases in the amount of this protein, is seen in some situations where there is an increase in its transcription (ie starvation, ER stress). The results obtained indicate that the levels of SQSTM1/p62 are elevated, but the autophagic flux is not impaired.

To assess the involvement of the ER stress in δ‐TT‐induced autophagy, PC3 cells were pretreated with salubrinal (20 μM, 4 hours) or 4‐PBA (2 mM, 1 hours), before treatment with δ‐TT (15 μg/mL, 24 hours). We showed that both ER stress inhibitors counteracted the tocotrienol‐triggered increase of the LC3‐II/LC3‐I ratio as well as that of SQSTM1/p62 expression (Figure 6D,E).

In conclusion, in PC3 cells (but not in autophagy‐defective DU145 cells), δ‐TT‐induced autophagy is related to the upstream activation of the ER stress pathways (ER stress‐autophagy axis).

### 3.7 Autophagy mediates the antitumour activity of δ‐TT in PC3 prostate cancer cells

To assess whether autophagy might mediate the proapoptotic activity of δ‐TT in PC3 cells, cells were pretreated with 3‐MA (10 mM, 4 hours) before δ‐TT (15 μg/mL, 24 hours). Caspase 3 and PARP cleavage and cell viability were investigated (Western blot and MTT assay). Figure 6F shows that δ‐TT increased the expression of cleaved caspase 3 and PARP, as previously observed. 3‐MA, given alone, did not affect the levels of these proteins; on the other hand, 3‐MA significantly counteracted the effects of δ‐TT on the expression levels of cleaved caspase 3 and PARP (Figure 6F).

As expected, cell viability was significantly suppressed by δ‐TT, while it was not affected by 3‐MA; however, the autophagy inhibitor significantly reverted (although not completely) the cytotoxic effect of δ‐TT (Figure 6G).

### 3.8 δ‐TT triggers paraptosis in prostate cancer cells

Data reported above suggest that δ‐TT may exert its activity by triggering non‐canonical pro‐death mechanisms in addition to apoptosis (see Figure 2C). Paraptosis represents an alternative cell death mechanism characterized by extensive vacuolation related to ER stress/mitochondria swelling.^18^, ^32^, ^33^ Paraptosis was also reported to be dependent on protein synthesis.^34^, ^35^

**Figure 7:**
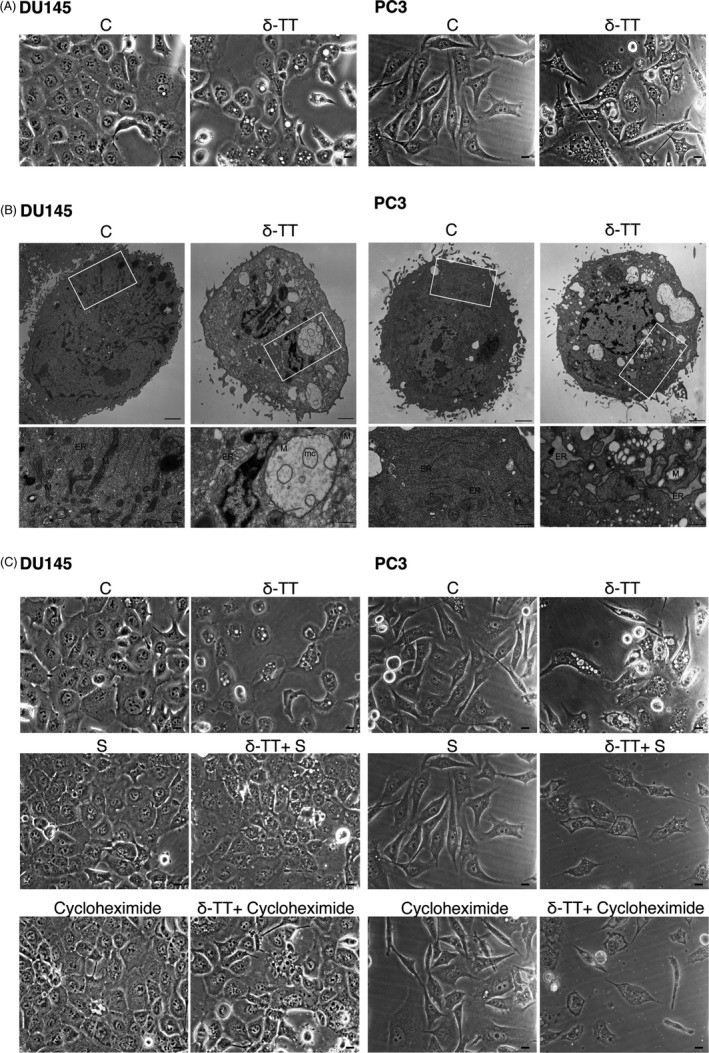
δ‐TT triggers paraptosis in prostate cancer cells. In all these experiments, DU145 and PC3 cells were treated with δ‐TT (15 μg/mL) for 18 h. A, Light microscopy highlighting the presence of extensive cytoplasmic vacuolation in treated cells. Scale bars are 20 μm. B, TEM micrographs showing the presence of swollen damaged mitochondria (M), with loss or disintegrated cristae (mc) and endoplasmic reticulum (ER) cisternae dilatation (boxed areas), in both DU145 and PC3 treated cells. Scale bars are 2 μm, and 0.7 μm for photographs in boxed areas. C, Light microscopy showing that pre‐treatment of DU145 and PC3 cells with either the ER stress inhibitor salubrinal (S; 20 μM) for 4 h, or the translation inhibitor cycloheximide (20 μM) for 3 h, markedly suppresses cytoplasmic vacuolation in δ‐TT‐treated prostate cancer cells. Scale bars are 20 μm

We found that δ‐TT (15 μg/mL, 18 hours) induces cytoplasmic vacuolation in both DU145 and PC3 cells (Figure 7A). By TEM analysis, we observed that untreated CRPC cells exhibit a normal appearance with normal mitochondria and ER with small profiles of cisternae. On the other hand, cells treated with δ‐TT showed the presence of swollen damaged mitochondria with loss/altered cristae, and ER cisternae dilatation (Figure 7B, boxed areas). Moreover, pre‐treatment of CRPC cells with the ER stress inhibitor salubrinal (20 μM, 4 hours) markedly suppressed δ‐TT‐induced cytoplasmic vacuolation (Figure 7C), supporting the relationship between vacuoles and ER stress. To confirm the involvement of paraptosis in the anti‐cancer activity of δ‐TT, DU145 and PC3 cells were pretreated with cycloheximide (20 μM, 3 hours) and then with the tocotrienol. Translation inhibition strikingly suppressed the cytoplasmic vacuolation induced by δ‐TT in both cell lines (Figure 7C).

**Figure 8:**
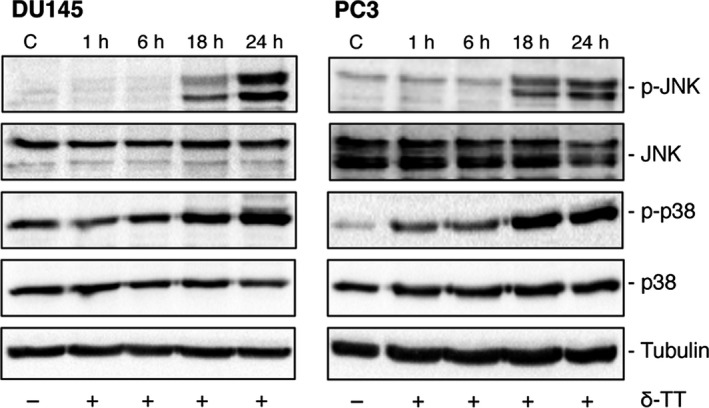
δ‐TT increases the expression of proteins involved in the MAPK cascade in prostate cancer cells. DU145 and PC3 cells were treated with δ‐TT (15 μg/mL) for 1‐24 h. p‐JNK and p‐p38 expression levels were evaluated by Western blot analysis. Tubulin expression was assessed as a loading control. One representative of three different experiments performed is shown

Finally, the effects of δ‐TT (15 μg/ml, 1‐24 hours) were analysed on the expression of MAPK proteins, known to be involved in paraptosis. δ‐TT (18 and 24 hours) increased the levels of both p‐JNK and p‐p38 kinases (Figure 8).

These data support that paraptosis is involved in the anti‐cancer activity of δ‐TT in prostate cancer cells.

## 4 DISCUSSION

It is accepted that TTs are associated with significant anti‐cancer properties.^24^, ^36^ Although most of the studies so far reported were performed with γ‐TT, δ‐TT was shown to be the most effective vitamin E isoform in triggering cell death in prostate cancer cells^37^, ^38^; however, the molecular mechanisms/targets of this activity are poorly clarified.

Here, we dissected the molecular mechanisms underlying the antitumour/proapoptotic activity of δ‐TT in CRPC cells (DU145 and PC3); the possible involvement of paraptosis in its activity was also investigated.

We confirmed that δ‐TT exerts a significant antitumour/cytotoxic activity on CRPC cells, by decreasing cell viability, increasing the dead/live cells ratio and reducing the viability of colony‐forming cells. Interestingly, the tocotrienol reduced the viability of RWPE‐1 cells only slightly and at the highest dose. We also demonstrated that δ‐TT triggers apoptosis by increasing the levels of cleaved caspase 3 and PARP and releasing cytochrome *c* from mitochondria into the cytoplasm. We finally confirmed the involvement of the intrinsic apoptosis in the activity of δ‐TT by showing that pre‐treatment of the cells with the pan‐caspase inhibitor Z‐VAD‐FMK significantly counteracts its cytotoxic effects.

These data agree with previous observations showing that δ‐TT induces cell death in prostate cancer cells^37^, ^38^ and suppresses the survival of the stem‐like cells subpopulation of PC3 cells^40^; similar results were reported for γ‐TT.^24^, ^41^, ^42^

In line with these data, the anti‐cancer activity of TTs (specifically γ‐ and δ‐TT) was reported in a wide range of tumours.^24^, ^45^, ^46^

To get further insights into the mechanisms and targets of the δ‐TT antitumour activity in CRPC cells, we concentrated our studies on the ER stress and autophagy pathways. We observed that, in both DU145 and PC3 cells, δ‐TT induces the expression of BiP, p‐eIF2α and IRE1α. δ‐TT also induced the expression/activation of the transcription factors ATF4 and CHOP (pointing out their cytoplasmic‐to‐nuclear localization). It is known that the p‐eIF2α/ATF4 pathway activates CHOP, a transcription factor that is also activated by IRE1α.^14^, ^51^ These results demonstrate that, in CRPC cells, δ‐TT triggers the main ER stress branches, leading to the activation of CHOP, deeply involved in the ER stress‐related apoptosis. To confirm the involvement of the ER stress pathway in the activity of δ‐TT, we pretreated the cells with two ER stress inhibitors, salubrinal and 4‐PBA. Both inhibitors significantly reverted the proapoptotic effect of δ‐TT, as assessed in terms of cleavage of caspase 3 and PARP as well as of cell viability, indicating that ER stress mediates its anti‐cancer activity.

We also investigated whether autophagy might be induced by δ‐TT in CRPC cells. First, we demonstrated that the tocotrienol markedly increases the expression of autophagy‐related proteins, such as LC3 (increased LC3‐II/LC3‐I ratio) and SQSTM1/p62 and their accumulation into autophagosomes in PC3, but not in DU145 cells (previously reported to be autophagy‐defective).^31^ Thus, the involvement of autophagy in the antitumour activity of δ‐TT was further investigated in PC3 cells. By TEM, we demonstrated the presence of autophagosomes and autophagolysosomes in δ‐TT‐treated cells. Pre‐treatment of the cells with an early stage autophagy inhibitor (3‐MA) significantly counteracted, while their pre‐treatment with late stage autophagy inhibitors (CQ and Baf) markedly increased δ‐TT‐induced LC3‐II and SQSTM1/p62 expression. These data support that δ‐TT triggers an autophagic flux in PC3 cancer cells. We further showed that in PC3 cells, the autophagy pathway is linked to ER stress, since pre‐treatment of the cells with the ER stress inhibitors markedly prevented δ‐TT‐induced LC3‐II and SQSTM1/p62 overexpression. Finally, we demonstrated that the autophagy inhibitor 3‐MA significantly counteracts the effects of δ‐TT on apoptosis markers as well as on cell viability.

These data demonstrate that, in CRPC cells possessing an efficient autophagy pathway, δ‐TT induces apoptosis by triggering the ER stress‐related pro‐death autophagy pathway. On the other hand, only the ER stress pathway is involved in the activity of δ‐TT in autophagy‐defective cells.

To our knowledge, this is the first report describing the involvement of the ER stress‐autophagy in the anti‐cancer activity of δ‐TT in prostate cancer cells. γ‐TT was shown to concurrently trigger ER stress and autophagy in inducing apoptosis in breast cancer cells.^52^, ^53^ TTs (specifically γ‐ and δ‐TT) were shown to induce apoptosis by triggering the ER stress branches in cervical cancer,^30^ breast cancer^54^ and melanoma cells.^29^ In line with these observations, both the ER stress and the autophagy pathways were reported to mediate the anti‐cancer activity of several natural compounds.^55^, ^56^

Here, we also observed that abrogation of apoptosis by the pan‐caspase inhibitor Z‐VAD‐FMK significantly, but not completely, reverted the cytotoxic effect of δ‐TT on CRPC cells. Thus, an additional programmed cell death modality might be involved in the activity of the tocotrienol. Paraptosis, necroptosis, mitotic catastrophe, anoikis were reported to be typical of apoptosis‐resistant tumour cells and to mediate the cytotoxic effects of anti‐cancer compounds.^18^, ^20^, ^33^ This makes these types of cell death a promising target for novel therapeutic strategies.^33^ Among them, paraptosis is characterized by: intense cytoplasmic vacuolation, correlated with ER stress and mitochondrial swelling/dilatation; de novo protein synthesis; involvement of JNK and p38 kinases.^18^, ^21^, ^33^ We observed that δ‐TT induces morphological changes, with an intense cytoplasmic vacuolation in both CRPC cells. In δ‐TT‐treated cells, we pointed out: by TEM, a significant swelling of mitochondria and dilatation of the ER cisternae; by light microscopy, a cytoplasmic vacuolation that was markedly inhibited in the presence of salubrinal or cycloheximide; by Western blot, increased expression of the active forms of JNK and p38. These data support that, in addition to apoptosis, the non‐canonical cell death paraptosis is involved in the antitumour activity of δ‐TT in CRPC cells. So far, the role of paraptosis‐like cell death in the cytotoxic activity of δ‐TT has been reported only in colon carcinoma cells.^62^, ^63^ On the other hand, different natural compounds (such as taxol) were shown to trigger this cell death in different cancer cells.^18^, ^64^, ^65^

These data demonstrate that, in CRPC cells, δ‐TT exerts an anti‐cancer activity by triggering both apoptosis, involving the ER stress‐autophagy axis, and paraptosis, providing novel mechanistic insights into this activity.

## CONFLICTS OF INTEREST

The authors declare that they have no conflicts of interest related to this article.

## AUTHOR CONTRIBUTIONS

MTT, trypan blue, colony formation, Western blot assays, figure preparations and data analysis: FF, RMM; immunofluorescence and light microscopy studies and statistically analysis of the data: MR, MM; purification of δ‐TT: GB; TEM analysis: PP, PS; study design, data collection and critical revision of the manuscript: MMM, PL; manuscript preparation: PL. All authors discussed the results and revised the manuscript.
